# Lipoprotein(a), Coronary Complexity, and Stent-Related Outcomes: Meta-Analytic Insights for the Interventional Cardiologist

**DOI:** 10.3390/jcm15093359

**Published:** 2026-04-28

**Authors:** Alberto Cereda, Marco Stracqualursi, Matteo Rocchetti, Margherita Mariani, Matteo Carlà, Antonio Gabriele Franchina, Matteo Carelli, Alessandro Sticchi, Mario Galli, Stefano Lucreziotti

**Affiliations:** 1 Interventional Cardiology Unit, Department of Cardiology, San Carlo Borromeo Hospital, ASST Santi Paolo e Carlo, 20153 Milan, Italy; 2 Department of Cardiology, ASST Lariana, 22100 Como, Italy; 3 Cardioangiologisches Centrum Bethanien (CCB), MVZ CCB Frankfurt und Main Taunus GbR, Markus Hospital, Wilhelm Epstein Strasse 4, 60431 Frankfurt am Main, Germany; 4 Department of Surgical, Medical, Molecular Pathology and Critical Care Medicine, University of Pisa, 56126 Pisa, Italy; 5 Department of Biomedical Sciences, Humanitas University, 20072 Pieve Emanuele, Italy; 6 IRCCS Humanitas Research Hospital, 20089 Rozzano, Italy; 7 EMO-GVM Centro Cuore Columbus, 20145 Milan, Italy

**Keywords:** lipoprotein(a), percutaneous coronary intervention, coronary artery disease complexity, restenosis, stent-related outcomes

## Abstract

**Background:** Lipoprotein(a) [Lp(a)] is an inherited cardiovascular risk factor, but its relationship with coronary anatomical complexity, plaque phenotype, and outcomes after percutaneous coronary intervention (PCI) remains incompletely defined. **Methods:** We conducted a systematic review and meta-analysis of studies evaluating the association between circulating Lp(a) levels and coronary disease characteristics, post-PCI clinical outcomes, stent-related adverse outcomes, and aortic valve disease. **Results:** Twenty-six studies were included. Elevated Lp(a) levels were associated with greater coronary anatomical complexity and a higher risk of major adverse cardiovascular events after PCI (HR 1.4, 95% CI 1.2–1.7). The strongest associations were observed for stent-related adverse outcomes, including restenosis (OR 3.23, 95% CI 2.2–4.8) and target vessel revascularization (OR 2.6, 95% CI 1.6–4.4). Higher Lp(a) levels were also associated with vulnerable plaque features and aortic valve calcification. **Conclusions:** Elevated Lp(a) is associated with greater coronary disease complexity and adverse outcomes after PCI. Elevated Lp(a) may represent a biological marker identifying high-risk patients and providing additional insight for personalized risk stratification and procedural decision-making in patients undergoing PCI.

## 1. Introduction

Lipoprotein(a) [Lp(a)] is a genetically determined lipoprotein particle that has emerged as a causal contributor to both atherosclerotic cardiovascular disease (ASCVD) and calcific aortic valve disease (CAVD). Evidence derived from genetic analyses, epidemiological investigations, and Mendelian randomization studies has consistently demonstrated a causal association between elevated Lp(a) levels and heightened cardiovascular risk.

Consequently, major scientific societies now consider Lp(a) a relevant inherited risk factor for cardiovascular disease [1,2]. Despite the well-established relationship between Lp(a) and long-term cardiovascular outcomes, its influence on the phenotypic expression of coronary artery disease and on vascular responses to mechanical injury remains incompletely characterized.

Beyond promoting lipid accumulation within the arterial wall, Lp(a) is involved in multiple biological processes that contribute to plaque vulnerability and defective vascular repair. Lp(a) carries a substantial burden of oxidized phospholipids and exhibits structural similarities with plasminogen through its apolipoprotein(a) component, thereby promoting endothelial dysfunction, vascular inflammation, oxidative stress, and antifibrinolytic activity [3,4,5]. Through these mechanisms, Lp(a) may accelerate the progression of atherosclerosis and increase thrombotic risk while impairing physiological vascular healing following percutaneous coronary intervention (PCI). Consequently, Lp(a) levels may influence long-term cardiovascular risk and stent-related outcomes such as restenosis, target lesion revascularization, and recurrent ischemic events. However, the current evidence remains inconsistent and is predominantly derived from observational studies [6,7,8].

Contemporary approaches to coronary risk stratification are predominantly centered on the detection of hemodynamically significant stenoses and the assessment of functional myocardial ischemia. However, evidence from invasive and non-invasive imaging studies has demonstrated that a substantial proportion of acute coronary events arise from non-flow-limiting lesions characterized by high-risk plaque features, including lipid-rich necrotic cores, thin-cap fibroatheroma, and large plaque burden [9,10,11]. These observations highlight the importance of biological mechanisms underlying plaque vulnerability that are not adequately reflected by ischemia-based evaluation alone.

In this context, Lp(a) may emerge as a cornerstone biomarker for understanding plaque biology and vulnerability, potentially linking functional assessment of ischemia and structural identification of high-risk plaque phenotypes observed from invasive and non- invasive imaging. Several observational investigations have reported associations between Lp(a) levels and high-risk plaque imaging features, as well as, increased atherosclerotic burden and coronary anatomical complexity [12,13,14].

However, prior investigations have typically evaluated clinical events, plaque morphology, and procedural outcomes in isolation, without integrating anatomical complexity, plaque phenotype, stent-related complications, and valvular calcification into a unified mechanistic framework.

By integrating evidence across these domains, we aimed to better characterize the vascular phenotype associated with elevated Lp(a) levels and to provide insights for risk stratification and procedural decision-making in patients undergoing coronary intervention.

## 2. Methods

### 2.1. Search Strategy

**Scheme 1:**
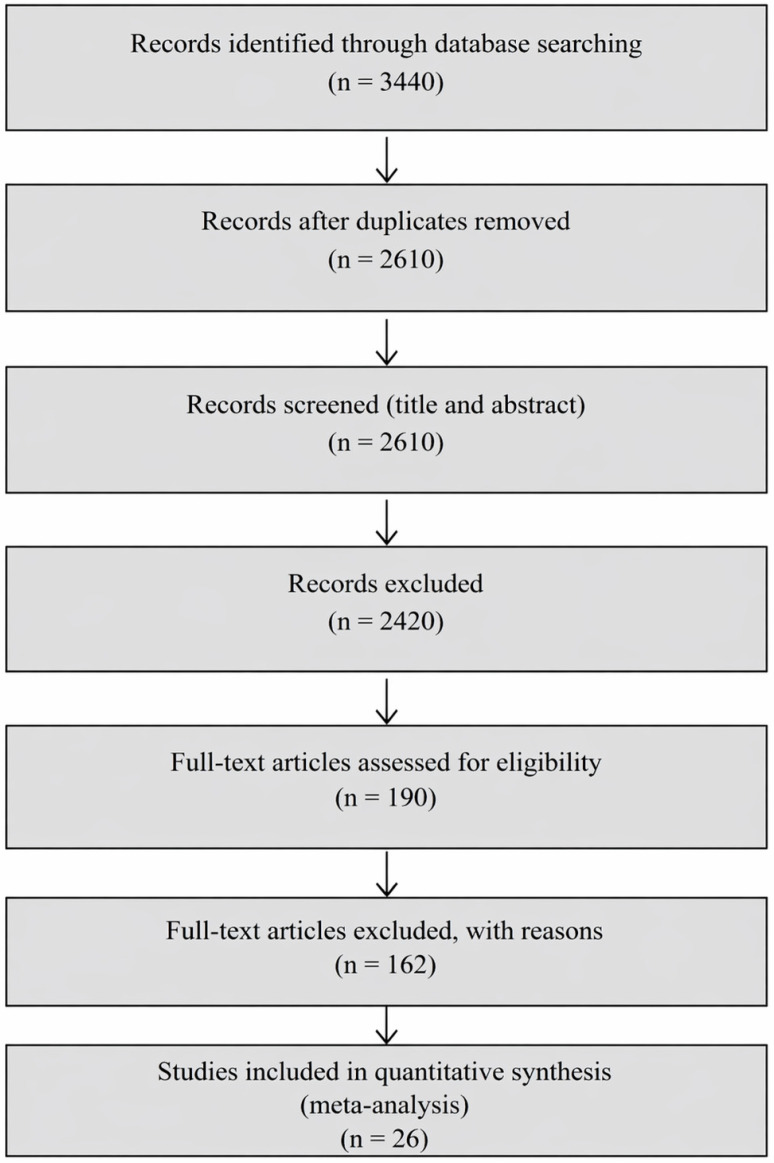
PRISMA flow diagram of study selection, resulting in 26 studies included in the quantitative synthesis.

A systematic literature search was conducted in PubMed/MEDLINE, Embase, and Web of Science from database inception until January 2025. The search strategy combined terms related to lipoprotein(a) with coronary artery disease, coronary imaging modalities, percutaneous coronary intervention, aortic valve disease, and cardiovascular clinical outcomes. No restrictions on publication date were applied. Only studies involving human subjects and published in English were considered (Scheme 1).

As reported in the flowchart, a total of 26 studies were included after full-text screening. The main reasons for exclusion (*n* = 162) were lack of relevant outcomes (*n* = 58), insufficient data or absence of adjusted estimates (*n* = 34), duplicate or overlapping populations (*n* = 21), non-original articles (*n* = 27), non-human or non-English studies (*n* = 16), and unclear or non-standardized Lp(a) assessment methods (*n* = 6).

The results initially yielded 3440 results; after removing duplicates, a total of 2610 articles were screened by title. Subsequently, 190 articles underwent abstract screening, and 162 articles were assessed for eligibility and underwent full-text review. Finally, 26 articles were selected for inclusion in this meta-analysis (see PRISMA flow diagram). Eligible studies included observational cohort studies, case–control studies, and post hoc analyses of randomized clinical trials reporting adjusted effect estimates. Outcomes were assessed at the longest available follow-up within each study. Only articles reporting at least one clinical endpoint of interest (see below) were selected. Outcomes were prespecified and analyzed separately to avoid conceptual overlap.

### 2.2. Data Extraction

Two independent investigators (AC and MS) screened titles and abstracts for eligibility, followed by full-text assessment of potentially relevant articles. Discrepancies were resolved by consensus discussion or consultation with a third reviewer. Data extraction was performed independently using a standardized data collection form. Extracted data included study characteristics, population features, methods of lipoprotein(a) assessment, outcome definitions, duration of follow-up, and adjusted effect estimates.

### 2.3. Population, Exposure, Comparator, Outcomes, and Study Design (PICOT Framework)

Population: adults with established/suspected coronary artery disease (undergoing imaging, PCI, or clinical follow-up) or aortic valve disease.

Exposure: circulating lipoprotein(a) [Lp(a)] levels assessed as continuous or categorical variables, without reclassification, according to the thresholds and definitions adopted in the original studies.

Comparator: incremental increases in Lp(a) concentration or higher versus lower concentration cohorts.

### 2.4. Outcomes

Prespecified outcomes were grouped into five distinct research domains:

1. Coronary anatomical complexity, including angiographic measures of disease burden.
2. Coronary plaque phenotype assessed by invasive or non-invasive imaging techniques.
3. Stent-related outcomes, including restenosis and repeat coronary revascularization.
4. Major adverse cardiovascular events during follow-up after coronary intervention.
5. Aortic valve calcification, hemodynamic progression, and valve-related clinical outcomes.

### 2.5. Statistical Analysis

Quantitative data are reported as mean values with standard deviation, and categorical variables are expressed in terms of percentages. Pooled effect estimates were calculated using random-effect models to account for between-study heterogeneity. Effect measures included odds ratios or hazard ratios for binary outcomes and standardized mean differences for continuous outcomes, each reported with corresponding 95% confidence intervals. Effect estimates were logarithmically transformed prior to pooling when appropriate.

Statistical heterogeneity was assessed using the I^2^ statistic with values of 25%, 50%, and 75% representing low, moderate, and high heterogeneity, respectively. Sensitivity analyses were performed by sequential exclusion of individual studies to evaluate the robustness of the pooled estimates. Studies contributing data to more than one outcome domain were included only once within each domain-specific analysis. The risk of bias of the included studies was evaluated independently by two investigators, taking into account study design, population selection, exposure assessment, outcome definition, and adjustment for potential confounders. Detailed risk of bias assessments for individual studies are reported in Tables S1 and S2. Publication bias was assessed visually using funnel plots when at least ten studies were available for a given outcome domain.

Given the heterogeneity in Lp(a) thresholds across studies, effect estimates were pooled as reported in the original studies (e.g., highest versus lowest categories or per unit increase), without reclassifying exposure levels. This approach preserves study-specific definitions and aligns with standard meta-analytic methodology. Sensitivity analyses restricted to studies using a ≥50 mg/dL threshold were not feasible due to limited data availability and heterogeneity in reporting.

Hazard ratios and odds ratios were pooled separately using random-effect models (DerSimonian and Laird method), after logarithmic transformation of effect estimates and corresponding standard errors.

## 3. Results

### 3.1. Association Between Lipoprotein(a) Levels and Coronary Anatomical Complexity

**Figure 1:**
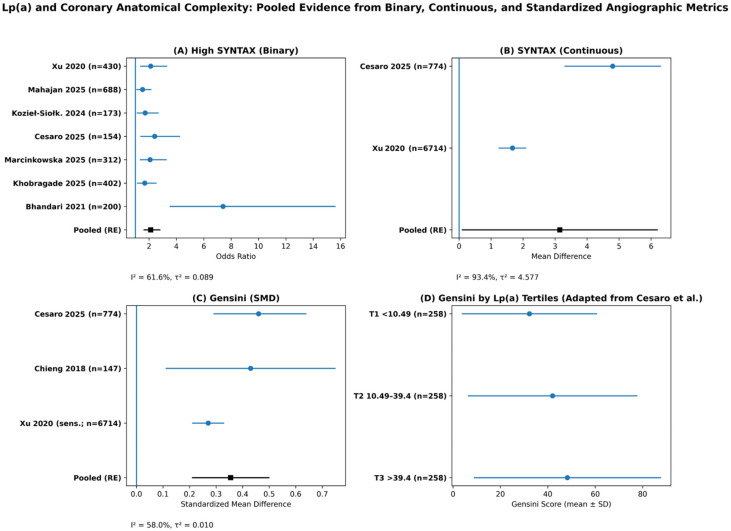
**Association between lipoprotein(a) levels and coronary anatomical complexity.** Forest plots showing the association between circulating lipoprotein(a) concentrations and angiographic measures of coronary artery disease burden. (**A**) Pooled odds ratio for the presence of high SYNTAX score (binary outcome) [1,2,3,4,5,6,7]. (**B**) Mean difference in SYNTAX score according to Lp(a) levels (continuous outcome) [1,4]. (**C**) Standardized mean difference in Gensini score between higher and lower Lp(a) groups [1,4,8]. (**D**) Mean Gensini score across increasing Lp(a) tertiles (adapted from Cesaro et al.). Blue dots represent individual study effect estimates, with horizontal blue lines indicating 95% confidence intervals. Black squares represent pooled effect estimates, with horizontal black lines indicating 95% confidence intervals. Random-effect models were used for all pooled analyses.

Across multiple angiographic metrics, elevated lipoprotein(a) levels were consistently associated with greater coronary anatomical complexity. In the binary analysis, pooling seven studies demonstrated that higher Lp(a) concentrations were associated with an approximately two-fold increased likelihood of presenting with a high SYNTAX score (I^2^ = 61.6%). In analyses using continuous measures of coronary disease burden, individuals with elevated Lp(a) exhibited significantly higher SYNTAX scores, with a pooled mean difference of approximately 3 points (I^2^ = 93.4%). Similarly, standardized mean difference analysis of the Gensini score across three studies showed that higher Lp(a) levels were associated with significantly greater angiographic disease burden (SMD 0.35; I^2^ = 58.0%). Consistent with these findings, tertile-based analysis demonstrated a stepwise increase in mean Gensini score across increasing Lp(a) categories, with patients in the highest tertile exhibiting the greatest degree of coronary atherosclerotic complexity (Figure 1).

### 3.2. Association Between Lipoprotein(a) Levels and Major Adverse Cardiovascular Events After PCI

**Figure 2:**
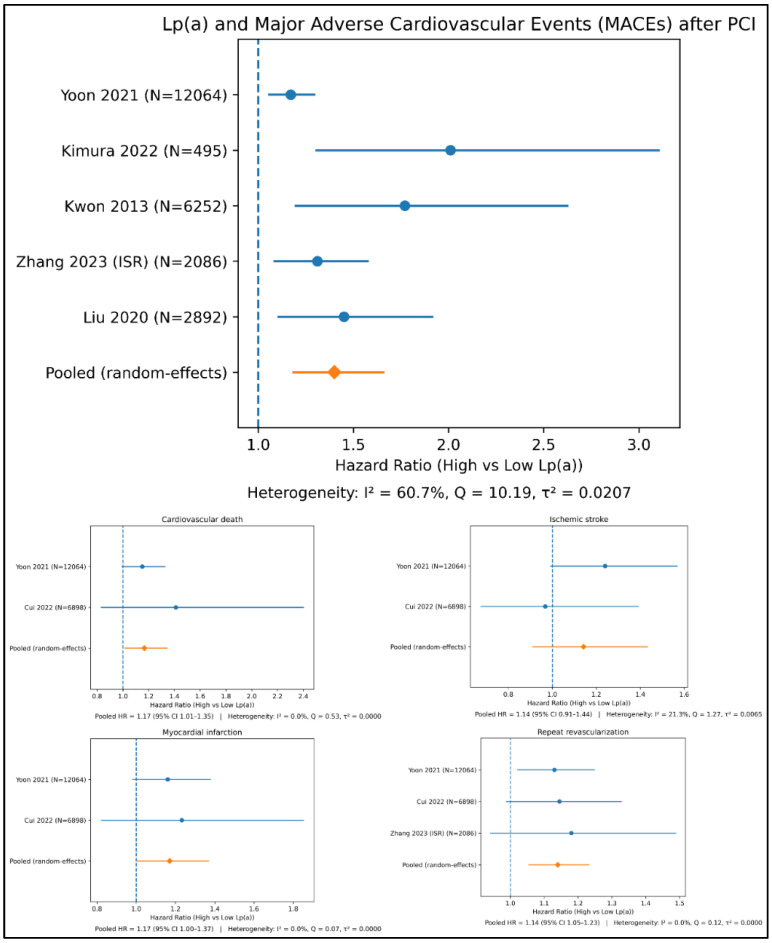
Association between lipoprotein(a) levels and major adverse cardiovascular events after percutaneous coronary intervention. Forest plots showing the association between circulating lipoprotein(a) concentrations and clinical outcomes following percutaneous coronary intervention, including major adverse cardiovascular events (MACE), cardiovascular death, ischemic stroke, myocardial infarction, and repeat revascularization [9,10,11,12,13,14]. Random-effects models were used for all pooled analyses [9,12,14]. Circles indicate study-specific hazard ratios, with horizontal lines representing 95% confidence intervals; the diamond represents the pooled estimate; the dashed vertical line indicates the line of no effect (HR = 1).

Elevated lipoprotein(a) levels were consistently associated with a higher risk of adverse clinical outcomes following percutaneous coronary intervention. In the pooled analysis of five studies, patients with higher Lp(a) concentrations experienced a significantly increased risk of post-PCI major adverse cardiovascular events (HR 1.4, 95% CI 1.2–1.7). This association extended across individual components of the composite endpoint, with elevated Lp(a) linked to a greater risk of cardiovascular death (HR 1.17, 95% CI 1.0–1.3) and myocardial infarction (HR 1.17, 95% CI 1.1–1.3), and a similar direction of effect observed for ischemic stroke (HR 1.14, 95% CI 1.0–1.3). Moreover, higher Lp(a) levels were significantly associated with an increased likelihood of repeat revascularization (HR 1.14, 95% CI 1.1–1.2), supporting a potential role of Lp(a) in promoting both recurrent ischemic events and progressive coronary disease following revascularization (Figure 2).

### 3.3. Association Between Lipoprotein(a) Levels and Stent-Related Outcomes

**Figure 3:**
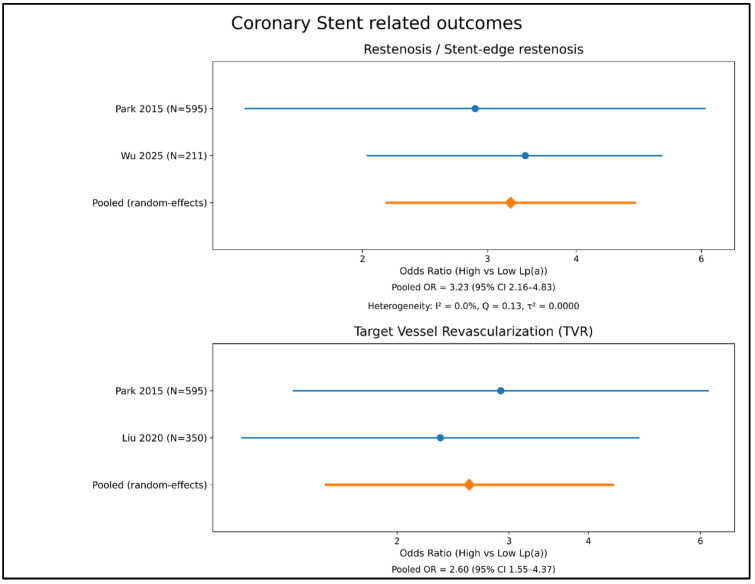
Association between lipoprotein(a) levels and stent-related outcomes after percutaneous coronary intervention. Forest plots showing the association between circulating lipoprotein(a) concentrations and adverse stent-related outcomes, including restenosis or stent-edge restenosis and target vessel revascularization (TVR). Random-effects models were used for all pooled analyses [13,15,16]. Colors are used for visual distinction only and do not convey additional analytical meaning.

After PCI Elevated lipoprotein(a) levels were significantly associated with an increased risk of adverse stent-related outcomes following percutaneous coronary intervention. In the pooled analysis, higher Lp(a) concentrations were associated with a more than three-fold increased risk of restenosis or stent-edge restenosis (OR = 3.23, 95% CI 2.16–4.83). Similarly, elevated Lp(a) levels were associated with a significantly increased risk of target vessel revascularization (OR = 2.60, 95% CI 1.55–4.37), suggesting a potential role of Lp(a) in promoting neointimal proliferation and recurrent vessel obstruction after coronary stenting (Figure 3).

### 3.4. Association Between Lipoprotein(a) Levels and Coronary Plaque Phenotype

**Figure 4:**
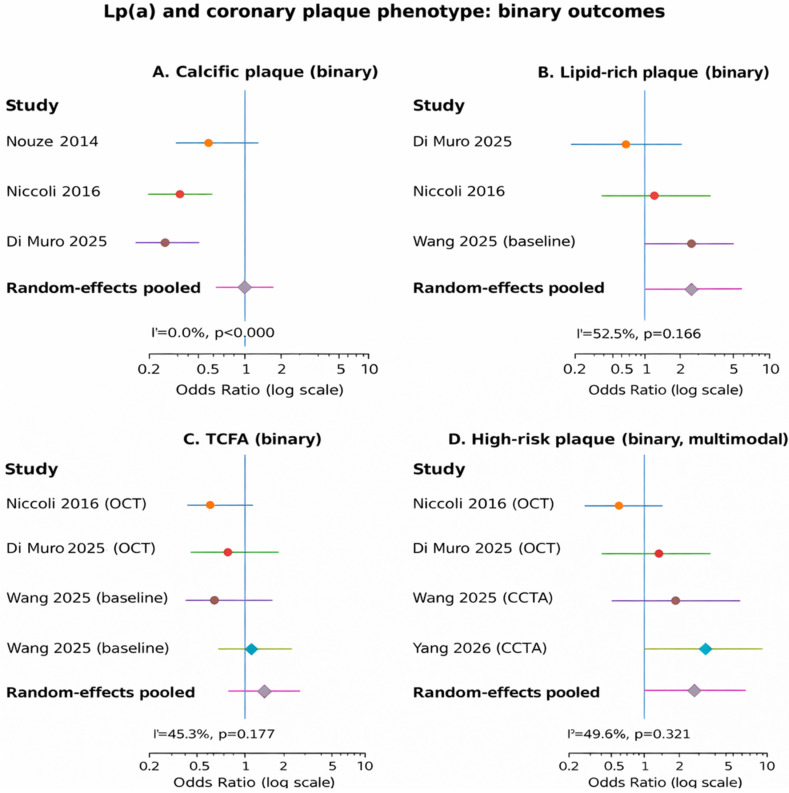
Association between lipoprotein(a) levels and coronary plaque phenotype. Forest plots showing the association between circulating lipoprotein(a) concentrations and coronary plaque characteristics, including calcific plaque, lipid-rich plaque, thin-cap fibroatheroma (TCFA), and high-risk plaque defined by multimodality imaging (optical coherence tomography or coronary computed tomography angiography). Random-effects models were used for all pooled analyses. Circles indicate study-specific odds ratios, with horizontal lines representing 95% confidence intervals; the diamond represents the pooled estimate; the vertical line indicates the line of no effect (OR = 1) [17,18,19,20,21].

Elevated lipoprotein(a) levels were differentially associated with specific coronary plaque phenotypes. In pooled analyses, higher Lp(a) concentrations were not significantly associated with the presence of calcific plaque (OR 0.9, 95% CI 0.7–1.2). In contrast, elevated Lp(a) levels showed a trend toward increased odds of lipid-rich plaque (OR 1.6, 95% CI 1.0–2.4) and thin-cap fibroatheroma (TCFA) (OR 1.5, 95% CI 0.9–2.5), although these associations did not consistently reach statistical significance across studies. When broader multimodality definitions of high-risk plaque were considered, including both optical coherence tomography and coronary computed tomography angiography-derived features, higher Lp(a) levels were associated with increased odds of high-risk plaque characteristics (OR 2.0, 95% CI 1.2–3.3), suggesting a potential role of Lp(a) in promoting vulnerable plaque morphology (Figure 4).

### 3.5. Association Between Lipoprotein(a) Levels and Aortic Valve Disease

**Figure 5:**
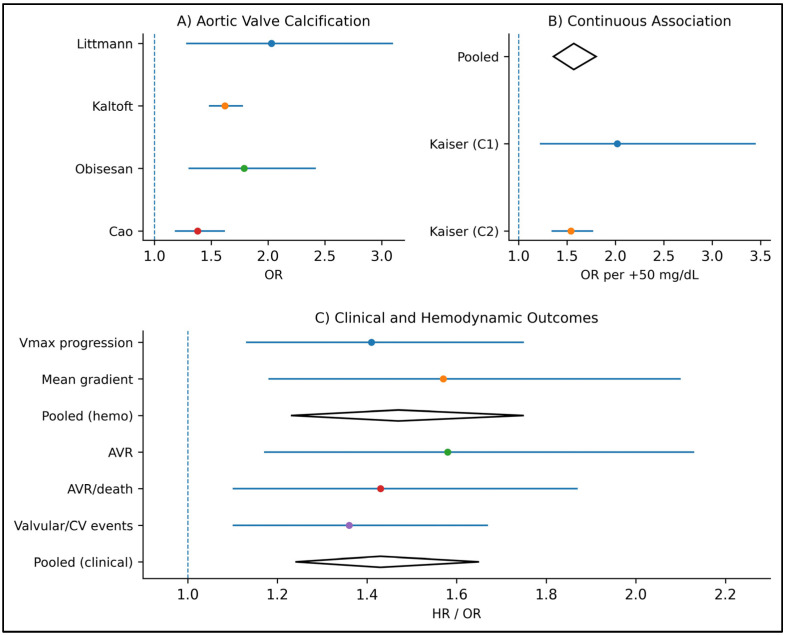
Association between lipoprotein(a) levels and aortic valve disease. Forest plots showing the association between circulating lipoprotein(a) concentrations and aortic valve calcification and clinical outcomes. (**A**) Presence of aortic valve calcification (categorical analysis) [22,23,24,25]. (**B**) Continuous association between Lp(a) levels and computed tomography–defined aortic valve calcification [26]. (**C**) Structured summary of clinical and hemodynamic outcomes, including aortic valve replacement and valvular or cardiovascular events. Random-effect models were used for all pooled analyses. Different colors are used to distinguish individual studies and do not represent different outcome categories or analytical groups.

Elevated lipoprotein(a) levels were consistently associated with the presence and progression of aortic valve calcification across categorical and continuous analyses. In pooled categorical analyses, higher Lp(a) concentrations were associated with increased odds of prevalent aortic valve calcification, with effect estimates ranging from approximately 1.4 to 2.0 across cohorts. In continuous analyses, each incremental increase in circulating Lp(a) was associated with a higher likelihood of computed tomography–defined aortic valve calcification, supporting a dose-dependent relationship between Lp(a) burden and valvular calcium deposition. Consistent with these findings, structured outcome analyses demonstrated that elevated Lp(a) levels were associated with hemodynamic progression of aortic stenosis and an increased risk of adverse clinical outcomes, including aortic valve replacement and valvular or cardiovascular events (Figure 5).

### 3.6. Differential Cardiovascular Impact of Elevated Lipoprotein(a) Levels

Across multiple anatomical and clinical endpoints, elevated lipoprotein(a) levels were associated with an increased risk of adverse cardiovascular outcomes. While higher Lp(a) concentrations were consistently linked to major adverse cardiovascular events, cardiovascular death, ischemic stroke, and the presence of high-risk plaque features, the magnitude of these associations was generally modest across most outcomes.

**Figure 6:**
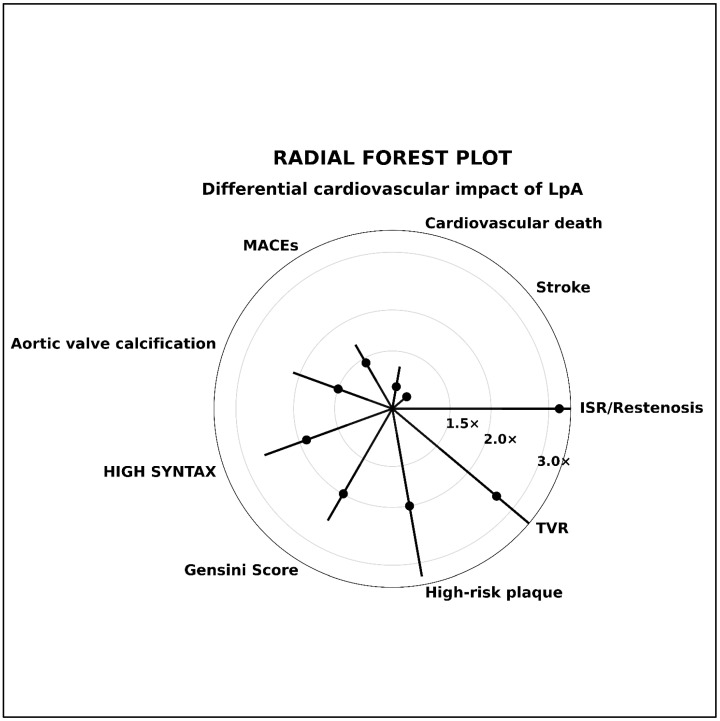
Differential cardiovascular impact of elevated lipoprotein(a) levels. Radial forest plot summarizing pooled effect estimates across anatomical and clinical cardiovascular outcomes derived from random-effect meta-analyses. The largest associations were observed for stent-related outcomes, including in-stent restenosis and target vessel revascularization.

Notably, the largest effect estimates were observed for stent-related outcomes. Elevated Lp(a) levels were associated with the highest pooled odds ratios for in-stent restenosis and target vessel revascularization, exceeding those observed for both clinical events and structural markers of coronary disease. These findings suggest that, beyond its established role in promoting atherosclerotic burden and plaque vulnerability, Lp(a) may exert a particularly relevant impact on neointimal proliferation and post-procedural vessel failure following coronary stenting (Figure 6).

## 4. Discussion

Elevated lipoprotein(a) levels showed a potential associated with increased coronary anatomical complexity across angiographic scoring systems in our meta-analysis. Higher Lp(a) concentrations were linked to a greater likelihood of presenting with high SYNTAX scores, as well as significantly higher continuous SYNTAX and Gensini values, suggesting a potential, even if not yet strongly defined, association with disease severity and extent rather than simple presence of coronary artery disease. These may also find some support in angiographic studies which can suggest progressively greater disease burden across SYNTAX categories and vessel involvement, with multivariate analyses hinting to Lp(a) as an independent predictor of coronary anatomical severity after adjustment for traditional risk factors. Evidence from the literature further indicates that this association may be influenced by clinical context, including elevated LDL cholesterol, diabetes, male sex, premature coronary disease, and specific ethnic groups such as South Asian populations, while variability in assay techniques and unit reporting may have contributed to heterogeneity across studies [1,3,6,7,8].

In this meta-analysis, elevated lipoprotein(a) levels are associated with an increased risk of adverse clinical outcomes following percutaneous coronary intervention, including major adverse cardiovascular events, cardiovascular death, myocardial infarction, ischemic stroke, and repeat revascularization. This is consistent with contemporary cohort and meta-analytic evidence linking elevated Lp(a) to higher risks of mortality, myocardial infarction, stroke, and repeat revascularization across cardiovascular populations [9,10,11,12]. In our analysis, the association is also suggested for repeat revascularization, theorizing a potential role of Lp(a) in progressive coronary disease and target lesion failure after PCI, possibly through impaired vascular healing and prothrombotic mechanisms [9,14].

The relationship between lipoprotein(a) and clinical outcomes after PCI remains heterogeneous across studies. Large cohorts demonstrated an association between elevated Lp(a) levels and recurrent ischemic events and MACE [9,10], while stent-focused studies linked higher Lp(a) to restenosis and device-related events [15,16]. In contrast, other cohorts did not confirm a consistent association with long-term outcomes despite a clear link with coronary disease severity [1]. This discrepancy likely reflects the multifactorial nature of post-PCI outcomes, including procedural factors, stent characteristics, and medical therapy, as well as heterogeneity in study design and follow-up. Overall, these findings suggest that Lp(a) primarily influences atherosclerotic phenotype and progression rather than directly predicting clinical events after PCI.

The present study is subject to several notable limitations. Several critical limitations must be carefully considered when interpreting the association between elevated Lp(a) levels and adverse post-PCI outcomes. Most notably, the significant heterogeneity reported across the included studies, alongside marked inconsistencies within specific subgroup analyses, complicates the generalizability of these findings. Furthermore, the notable absence of formal publication bias assessments in major meta-analyses raises the possibility that the current literature might overstate the true strength of this association. Consequently, these methodological constraints warrant a cautious application of these results to broader clinical practice.

Elevated lipoprotein(a) levels were associated with stent-related outcomes in our pooled analysis, including restenosis and target vessel revascularization; this may suggest a potential role of Lp(a) in neointimal proliferation and impaired vascular healing following PCI [15,16]. The greater magnitude of association observed for stent-related complications compared with broader clinical endpoints supports a preferential effect of Lp(a) on local vascular responses to injury rather than systemic atherosclerotic risk alone.

However, significant heterogeneity across studies should be considered. Ethnicity has been identified as a major contributor, with stronger associations reported in Asian populations. Variability in Lp(a) measurement techniques and threshold definitions, differences in stent technology across study eras, and incomplete angiographic follow-up may have introduced methodological and selection bias. Furthermore, variability in coronary imaging strategies and limited use of intravascular modalities may have influenced restenosis detection, contributing to inconsistencies in reported associations.

Elevated lipoprotein(a) levels have been consistently associated with high-risk coronary plaque features across multiple imaging-based studies [17,18,19,20]. Meta-analytic evidence suggests increased odds of vulnerable plaque characteristics, including thin-cap fibroatheroma and lipid-rich plaque morphology, in patients with elevated Lp(a) concentrations. Intravascular imaging studies using optical coherence tomography have reported higher prevalence of lipidic and TCFA-like features among patients with elevated Lp(a), while coronary computed tomography angiography studies have demonstrated an association between higher Lp(a) levels and accelerated progression of low-attenuation plaque components. Similar associations have also been reported in carotid plaque studies.

However, the available literature is limited by relatively small sample sizes and methodological heterogeneity, including differences in plaque definitions, imaging modalities, and patient populations. Significant heterogeneity has been reported in pooled analyses, alongside potential publication bias. Variability in imaging strategies, including the use of OCT versus coronary CT angiography, may also influence plaque characterization and contribute to inconsistencies in reported associations.

Elevated lipoprotein(a) levels are also linked to the progression of aortic valve calcification across pooled analyses, with higher concentrations suggesting higher odds of prevalent disease and a dose-dependent relationship with computed tomography–defined valvular calcium burden [22,23,24,25,26,27,28]. Elevated Lp(a) has also been associated with coronary artery calcification prevalence and progression, although findings across cohorts have been less consistent for cross-sectional CAC measures compared with longitudinal progression. Observational evidence further supports an association between elevated Lp(a) and the development and progression of calcific aortic valve disease, with Lp(a) recognized as a genetic risk factor for aortic stenosis [23,26,27].

However, the available literature is subject to several potential sources of bias, including retrospective study designs, variability in Lp(a) measurement and standardization, and differences in sample processing across cohorts. Imaging-related variability may also influence calcification assessment, with differences between cross-sectional CT-based measurements and longitudinal progression analyses potentially contributing to inconsistent findings. Heterogeneity in imaging protocols, calcium quantification methods, and the use of advanced imaging modalities such as PET or MRI may have further affected the characterization of vascular and valvular calcification across studies.

In this systematic review and meta-analysis, elevated Lp(a) levels were suggestive of a greater coronary anatomical complexity, adverse plaque characteristics, and post-PCI clinical outcomes, with the largest effect sizes observed for stent-related complications.

Importantly, the contemporary paradigm of coronary risk stratification remains largely centered on the identification of flow-limiting stenoses and functionally significant ischemia. However, a growing body of imaging-based evidence has demonstrated that adverse cardiovascular events frequently arise from non-flow-limiting lesions characterized by high-risk plaque features such as lipid-rich necrotic cores or thin-cap fibroatheroma. In this context, an ischemia-centered approach alone may fail to fully capture the biological activity of atherosclerotic disease, in a manner conceptually analogous to treating anemia in cancer without addressing the underlying malignancy [9,11]. Elevated Lp(a) levels may therefore provide incremental pathophysiological insight into plaque vulnerability, potentially helping to explain residual risk in patients undergoing ischemia-guided revascularization.

The preferential association observed between elevated Lp(a) levels and stent-related outcomes may reflect a localized effect on vascular healing following mechanical injury. Structurally, apolipoprotein(a) shares significant homology with plasminogen, potentially conferring antifibrinolytic properties that impair fibrin degradation and delay endothelial recovery after PCI. In addition, Lp(a)-associated pro-inflammatory and pro-oxidative pathways have been implicated in smooth muscle cell proliferation and neointimal hyperplasia, which represent key determinants of late lumen loss and target lesion failure. These mechanisms may contribute to an altered biological response to coronary stenting, supporting the hypothesis that Lp(a) modulates post-procedural vascular remodeling beyond its established role in systemic atherosclerotic risk [3,4,5].

From a translational perspective, elevated Lp(a) levels may help identify patients in whom plaque morphology and post-procedural vascular healing are more relevant determinants of outcome than ischemia severity alone [17,18,19]. In such individuals, imaging-guided PCI strategies aimed at optimizing stent expansion and lesion preparation, as well as intensified secondary prevention, may be particularly relevant to mitigate the risk of target lesion failure. These findings suggest that Lp(a) could contribute to a more refined risk stratification framework integrating functional, anatomical, and biological markers in patients undergoing coronary intervention. These observations may have relevant implications in the context of an increasingly personalized approach to lipid management. Ongoing development of targeted Lp(a)-lowering therapies, including antisense oligonucleotide and small interfering RNA agents, offers the potential to modulate disease activity beyond traditional LDL-cholesterol reduction. Whether selective Lp(a) lowering may improve post-PCI vascular healing and reduce the risk of target lesion failure remains to be determined in prospective imaging-integrated studies.

### Study Limitations

While this meta-analysis integrates anatomical complexity, plaque phenotype, stent-related outcomes, and valvular calcification within a unified multimodality framework, several limitations should be acknowledged. Most included studies were observational in design, limiting causal inference and exposing pooled estimates to potential selection bias and residual confounding. In addition, the use of pooled study-level data rather than individual patient-level data limited the ability to account for important clinical and procedural modifiers, including ethnicity, lipid profile, stent platform, and the use of intravascular imaging during PCI. Variability in Lp(a) measurement techniques and reporting units across studies may have influenced exposure classification and effect size estimation. Furthermore, coronary plaque phenotype was assessed using different imaging modalities, including optical coherence tomography and coronary computed tomography angiography, with non-uniform definitions of high-risk plaque. Finally, stent-related outcomes were derived from studies conducted across different technological eras, with variability in implantation techniques and post-procedural pharmacotherapy.

## 5. Conclusions

Elevated Lp(a) may be linked with greater coronary complexity and a higher risk of stent-related complications following PCI. The preferential association with restenosis and target vessel failure suggests a role in post-procedural vascular healing beyond systemic ischemic risk. Incorporating Lp(a) into risk stratification may support more personalized revascularization and lipid-lowering strategies.

This meta-analytic review suggests that lipoprotein(a) acts as a driver of an aggressive atherosclerotic phenotype, characterized by increased disease burden and features of plaque vulnerability, while its translation into clinical events remains heterogeneous.

## Data Availability

The data supporting the findings of this study are derived from previously published studies, which are cited within this manuscript. No new datasets were generated. Extracted data and analysis files are available from the corresponding author upon reasonable request.

## Supplementary Materials

The following supporting information can be downloaded at: https://www.mdpi.com/article/10.3390/jcm15093359/s1, Supplementary Table S1. Risk of bias assessment across the 26 included studies, stratified by research domain, showing overall low-to-moderate risk profiles; Supplementary Table S2. Characteristics of the included studies (*n* = 26), stratified by research domain, including design, population, endpoints, follow-up duration, and Lp(a) assessment, including PRISMA checklist table [29].
