# Supplementary material for: Lipoprotein(a), Coronary Complexity, and Stent-Related Outcomes: Meta-Analytic Insights for the Interventional Cardiologist
Source: J Clin Med. 2026 Apr 28;15(9):3359. doi: 10.3390/jcm15093359 (PMC13164280; doi:10.3390/jcm15093359)
Supplement: Supplementary file 1 [file jcm-15-03359-s001.zip › jcm-4263201-supplementary-table.pdf]

Supplementary Table S1. Risk of bias assessment across the 26 included studies, stratified by research domain, showing overall low-to-moderate risk profiles.

Supplementary Table S1. Risk of Bias Assessment (n = 26)

Research Domain	Study	Risk of Bias
Coronary Complexity	Xu (2019)	Low
	Mahajan (2025)	Low
	Koziel (2024)	Low
	Cesaro (2024)	Low
	Marcinkowska (2025)	Low
	Khobragade (2025)	Moderate
	Bhandari (2021)	Moderate
	Chieng (2018)	Low
MACE	Yoon (2021)	Low
	Kimura (2022)	Moderate
	Kwon (2013)	Moderate
	Zhang (2023)	Low
	Liu (2020)	Low
Stent Outcomes	Park (2015)	Low
	Wu (2025)	Low
	Liu (2020)	Low
Plaque Phenotype	Nozue (2014)	Low
	Niccoli (2016)	Moderate
	Di Muro (2024)	Low
	Wang (2025)	Low
	Yang (2025)	Low
Aortic Valve	Littman	Low
	Kaltroft	Moderate
	Obisesan	Moderate
	Cao (2016)	Moderate
	Kaiser (2021) – Amsterdam	Low
	Kaiser (2021) – Rotterdam	Low

Supplementary Table S2. Characteristics of the included studies (n = 26), stratified by research domain, including study design, population, outcomes, follow-up duration, and lipoprotein(a) assessment.

First Author	Year	Study Design	Population	N	Outcome(s)	Follow-up (months)
Xu	2020	Cross-sectional	Stable CAD	6714	SYNTAX score	NA
Mahajan	2025	Cross-sectional	ACS	NA	Coronary complexity	NA
Koziel-Siolkowska	2024	Cross-sectional	CAD	NA	SYNTAX score	NA
Cesaro	2025	Cohort	ACS	NA	CAD severity	NA
Marcinkowska	2025	Cross-sectional	Premature CAD	162	SYNTAX score	NA
Khobragade	2025	Cohort	CAD	NA	Angiographic severity	NA
Bhandari	2021	Cross-sectional	AMI	NA	CAD severity	NA
Chieng	2018	Cross-sectional	Premature CAD	NA	Lesion complexity	NA
Yoon	2021	Cohort	PCI	12064	CV death, MI, stroke	89
Kimura	2022	Cohort	PCI (DES)	495	MACE	36
Kwon	2013	Cohort	CAD	NA	Clinical outcomes	37
Zhang	2023	Cohort	PCI	2086	MACE	36
Liu	2020	Cohort	PCI	NA	Clinical outcomes	29
Cui	2022	Cohort	PCI	NA	Cardiovascular outcomes	NA
Park	2015	Cohort	PCI (DES)	595	ISR, outcomes	36
Wu	2025	Cohort	PCI	211	DoCE, TLR	24
Nozue	2014	Imaging study	CAD	NA	Lipid core progression	NA
Niccoli	2016	Imaging study	CAD	500	Plaque phenotype	NA
Di Muro	2025	Imaging study	ACS	NA	OCT plaque	NA
Wang	2025	Imaging study	ACS	NA	Non-culprit plaque	NA
Yang	2026	Imaging study	CAD	NA	PCAT/CCTA	NA
Wodaje	2022	Cohort	General population	NA	Aortic stenosis	168
Kaltoft	2022	Cohort	General population	NA	Aortic valve disease	NA
Obisesan	2022	Imaging study	General population	NA	Calcification	NA
Cao	2016	Cohort	Multi-ethnic population	NA	Aortic valve calcification	NA

Kaiser	2022	Cohort	General population	NA	Aortic valve calcification	NA

Abbreviations: CAD = coronary artery disease; ACS = acute coronary syndrome; AMI = acute myocardial infarction; PCI = percutaneous coronary intervention; DES = drug-eluting stent; MACE = major adverse cardiovascular events; ISR = in-stent restenosis; DoCE = device-oriented composite endpoint; TLR = target lesion revascularization; OCT = optical coherence tomography; CCTA = coronary computed tomography angiography; PCAT = pericoronary adipose tissue; NA = not available.
